# Impact of SGLT2 Inhibitors on Multiple Cardiometabolic Risk Factors: A Retrospective Cohort Study

**DOI:** 10.3390/jcm15072550

**Published:** 2026-03-27

**Authors:** Hilal Işık, Kader Kübra Demirdöğen, Teoman Çakır, Şevki Çetinkalp, Zeliha Kerry, Mehmet Zuhuri Arun

**Affiliations:** 1 Department of Clinical Pharmacy, İzmir Atatürk Training and Research Hospital, 35360 Izmir, Turkey; 2 Department of Clinical Pharmacy, Faculty of Pharmacy, Hacettepe University, 06230 Ankara, Turkey; 3 Division of Endocrinology and Metabolism Disorders, İzmir Atatürk Training and Research Hospital, 35150 Izmir, Turkey; 4 Division of Endocrinology and Metabolism Disorders, Faculty of Medicine, Ege University, 35100 Izmir, Turkey; 5 Department of Pharmacology, Faculty of Pharmacy, Ege University, 35100 Izmir, Turkey; 6 Department of Clinical Pharmacy, Faculty of Pharmacy, Ege University, 35100 Izmir, Turkey

**Keywords:** SGLT2Is, T2DM, cardiometabolic parameters

## Abstract

**Background:** Our study retrospectively investigated the therapeutic effects of SGLT2 inhibitors on multiple outcomes in patients with Type 2 Diabetes, capitalizing on the agent’s proven benefits in glycemic, cardiovascular, and renal systems. **Methods:** This retrospective cohort study investigated a total of 200 patients with T2DM, 100 SGLT2I-treated and 100 treated without SGLT2Is. Clinical data were retrieved from the electronic health record system of the hospital. Patients were followed for more than 6 months to assess the effects of SGLT2Is on metabolic, biochemical, and renal parameters. **Results:** In the SGLT2I-treated cohort, a higher prevalence of males, non-geriatrics, and comorbidities such as HF and ASCVD was observed with greater use of concomitant medications (beta-blockers, antithrombotics, antilipidemics). SGLT2I treatments show a greater reduction in FBG (control: −6.3 mg/dL vs. treatment: −24.2 mg/dL; *p* ≤ 0.05), HbA1c (control: −0.093% vs. treatment: −0.76%; *p* ≤ 0.001), weight (control: −0.6 kg vs. treatment: −3.6 kg; *p* ≤ 0.001), SBP (control: 5.8 mmHg vs. treatment: −9.2 mmHg; *p* ≤ 0.001), and DBP (control: 2.2 mmHg vs. treatment: −4.7 mmHg; *p* ≤ 0.05) compared to the control group. The analysis of the mean change in eGFR showed no statistically significant difference in both groups. The SGLT2I’s safety profile was favorable, with no difference in adverse events and no cases of euglycemic ketoacidosis or Fournier’s gangrene. **Conclusions:** In this study, SGLT2Is demonstrated strong clinical efficacy in improving multiple cardiometabolic parameters without compromising patient safety in short-term follow-up. Large-scale and long-term real-world studies are needed to monitor the long-term safety profile, characterize the incidence of rare adverse events in general clinical practice, and validate results from this study.

## 1. Introduction

In recent years, the prevalence of diabetes has increased worldwide due to the aging of the world’s population and the widespread rise in obesity. According to the 2021 IDF (International Diabetes Federation) data, 537 million people between the ages of 20 and 79 are living with diabetes. This number is projected to increase to 643 million in 2030 and 783 million in 2045 [1]. Various macrovascular and microvascular complications are the major causes of early morbidity and mortality associated with long-term poorly controlled T2DM (Type 2 Diabetes Mellitus), which also substantially increases healthcare costs [2,3].

Sodium glucose co-transporter 2 inhibitors (SGLT2Is) are a new generation of oral antidiabetic class used in the treatment of T2DM. Their mechanism of action involves the inhibition of the SGLT2 protein in the renal proximal tubule. This action results in reduced renal glucose reabsorption, thereby increasing urinary glucose excretion and ultimately lowering plasma glucose levels independently of insulin [4]. Owing to this insulin-independent mechanism of action, SGLT2 inhibitors are effective in patients regardless of their degree of insulin resistance or β-cell function. This feature minimizes the risk of hypoglycemia and allows them to be used effectively as monotherapy or in combination with other antidiabetic agents [4,5].

Treatment with SGLT2 inhibitors has been associated with sustained reductions in systolic and diastolic blood pressure [6]. This blood pressure-lowering effect is thought to primarily result from the sustained natriuretic and osmotic diuretic effects of the SGLT2Is. Furthermore, the accompanying glycosuria leads to caloric loss, which contributes to reductions in body weight and BMI [7].

SGLT2Is have also been shown to be associated with a reduction in the risk of major adverse cardiovascular events (MACEs) and to protect against cardiovascular death and death from any cause [8,9]. Furthermore, the EMPEROR-Reduced trial expanded the use of empagliflozin in the management of Heart Failure with Reduced Ejection Fraction (HFrEF), solidifying its role beyond just glucose control [10].

On the other hand, studies investigating the effects of SGLT2 inhibitors on renal events have shown that this group of drugs significantly reduces end-stage renal disease, doubling of serum creatinine level, and risk of renal death. These drugs have been associated with a reduction in risk factors such as macroalbuminuria, microalbuminuria, and worsening of nephropathy [11,12].

Consequently, current guidelines from major international societies, including the 2023 ESC [13,14], ADA [15], and KDIGO [16] guidelines, strongly advocate for the use of SGLT2 inhibitors across a wide spectrum of cardio-renal diseases. According to the 2023 ESC guidelines, SGLT2 inhibitors carry a Class I recommendation for patients with T2DM who have established atherosclerotic cardiovascular disease, heart failure, or chronic kidney disease, to effectively reduce cardiovascular and renal risks. These agents are now a cornerstone therapy for all phenotypes of heart failure, and they are the preferred agent for patients with T2DM and chronic kidney disease (eGFR ≥ 20 mL/min/1.73 m^2^ and above) to reduce the risk of end-stage kidney disease and major cardiovascular events. Importantly, these cardioprotective and renoprotective benefits apply regardless of the patient’s baseline glycemic control or concurrent glucose-lowering medications, extending the role of SGLT2 inhibitors well beyond glucose management [13,14].

This study aims to determine the differences between SGLT2 inhibitors and standard therapies in the treatment of T2DM over at least a 6-month period, focusing on their effects on body weight, blood sugar control, renal function, lipid profile, and adverse event profile.

## 2. Methods

### 2.1. Study Design

This retrospective cohort study was conducted in the endocrine outpatient clinic of Ege University Hospital, a tertiary care teaching and research hospital in Türkiye. This study was approved by the Ege University Ethics Committee on 7 October 2021 (approval number: 21-10T/9) and was conducted in accordance with the principles of the Declaration of Helsinki.

### 2.2. Patient Selection

Adult patients with a T2DM diagnosis who were followed for more than 6 months in the endocrinology outpatient clinic between 1 January 2021 and 1 October 2021 were identified. Pregnant or breastfeeding women and patients with T1DM were excluded from this study. Subsequently, patients receiving at least one oral antidiabetic medication were selected in this study. The cohort was stratified into two groups: the treatment group (patients on SGTL2 inhibitors) and the control group (patients on other antidiabetic regimens).

**Figure 1:**
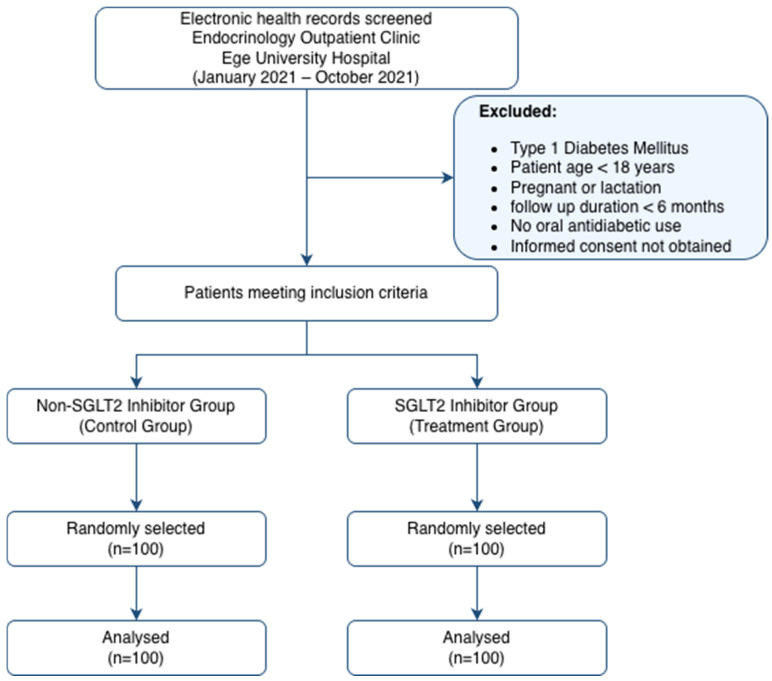
Study flow chart.

This study included adult patients (age ≥ 18 years) with a confirmed diagnosis of T2DM who were followed at the endocrinology outpatient clinic for at least six months between 1 January 2021 and 1 October 2021. Inclusion criteria required the use of at least one oral antidiabetic agent, provision of informed consent, and availability of consistent electronic medical records. Patients with T1DM, pediatric patients, pregnant or breastfeeding women, and those who declined participation were excluded. The cohort was divided into a treatment group (SGLT2 inhibitors) and a control group (other regimens). Since the eligible population exceeded the required sample size, 100 patients from each group were selected using simple random sampling (Figure 1).

### 2.3. Data Collection

Comprehensive clinical and laboratory data were extracted from the electronic medical records, including demographic characteristics (age, sex, education level), anthropometric measurements (height, weight, BMI, waist circumference), comorbid conditions (e.g., myocardial infarction, heart failure, renal and hepatic impairment), laboratory and vital parameters (e.g., blood pressure, average glucose, HbA1c, lipid profile, kidney function tests, and urinalysis results), and medication history (including all prescribed and over-the-counter medications and details regarding SGLT2 inhibitor use and associated side effects). All relevant information was systematically captured and entered into pre-designed, standardized Case Report Forms (CRFs). An SQL database (PostgreSQL) was created, with a specific drug table built using the registered drug list from the Turkish Medicines and Medical Devices Agency. Patient medication data were recorded via custom-developed software (developed by M.A.) and retrieved using SQL queries for statistical analysis.

### 2.4. Definition of Main Outcomes

The primary outcome was defined as the mean change in HbA1c level (%) measured between the baseline (initiation of SGLT2 inhibitor therapy) and the final follow-up visit.

Secondary outcomes included changes between baseline and the final follow-up visit in BMI, blood pressure, lipid profile, eGFR, and the incidence and types of adverse events.

### 2.5. Statistical Analysis

An a priori power analysis was performed using the G*Power software (version 3.1). Based on the HbA1c changes reported in the systematic review by Scheen [17] and assuming a power of 80% with an alpha error of 0.05, it was determined that 91 patients were required per group. To account for potential data attrition, the final sample size was set at 100 patients per group.

The normality of continuous data was assessed using the Kolmogorov–Smirnov test. Continuous variables were expressed as mean ± standard deviation (SD). For inter-group comparisons, the independent samples *t*-test was used for parametric data, while the Mann–Whitney U was employed for non-parametric data. For intra-group comparisons, the paired samples *t*-test and the Wilcoxon signed rank test were used for parametric data and non-parametric data, respectively. Categorical variables were analyzed using the Chi-square test and reported as counts and percentages. All these statistical analyses were performed using SPSS software (version 25.0), and a *p*-value ≤ 0.05 was considered statistically significant.

To address potential confounding from baseline group differences, multivariable linear regression analyses were performed for all primary continuous outcomes, with covariates selected based on clinical relevance. For HbA1c, an additional sensitivity analysis was performed using percentage change as the outcome variable. Heteroscedasticity-robust standard errors (HC3) were applied throughout. These supplementary analyses and post hoc power calculations were performed using the statsmodels library in Python (version 3.12).

## 3. Results

### 3.1. Study Population

A total of 200 patients were included in the study cohort, comprising 100 patients treated with SGLT2I (treatment group) and 100 patients not receiving SGLT2I (control group). A total of 44.5% of our cohort was male, and 27.5% was older than 65 years of age. The mean age was statistically significantly lower in the treatment group compared to the control (*p* ≤ 0.05). Furthermore, the treatment group had a significantly lower proportion of geriatric patients (≥65 years) and females compared to the control (*p* ≤ 0.05 and *p* ≤ 0.01, respectively). In this cohort, the treatment group showed a significantly higher prevalence of established atherosclerotic cardiovascular disease (ASCVD) compared to the control group (*p* ≤ 0.001). Specifically, the rates of patients diagnosed with coronary artery disease (CAD), a history of myocardial infarction (MI), and heart failure (HF) were also significantly greater in the treatment group (*p* ≤ 0.001 for all). The patients treated with SGLT2Is had significantly higher baseline HbA1c and FPG levels compared to the control group (*p* ≤ 0.001, *p* ≤ 0.05, respectively). Table 1 describes the baseline characteristics of the treatment and control groups.

Among patients in the treatment group, the majority received (81%) empagliflozin, while the remaining 19% received dapagliflozin. In a sub-group analysis based on the type of SGLT2I, patients treated with empagliflozin had a significantly higher prevalence of ASCVD compared to those treated with dapagliflozin (44.4% vs. 15.8%, *p* ≤ 0.05). Regarding formulation types within the empagliflozin group (*n* = 81), 35.8% of patients (*n* = 29) were treated with a fixed-dose combination (empagliflozin plus metformin), while the remaining 64.2% (*n* = 52) received empagliflozin as a single agent. However, when total metformin use was analyzed (both fixed-dose and separate co-administration), 86.4% (*n* = 70) of empagliflozin-treated patients were receiving metformin therapy.

The utilization of lipid-lowering medications, beta-blockers (BBs), and antithrombotic therapy was statistically significantly higher in the treatment group compared to the control group (*p* ≤ 0.01 for all) (Table 2). The most commonly prescribed lipid-lowering medication was atorvastatin, and the most frequently used antithrombotic was acetylsalicylic acid (*n* = 70 and *n* = 56, respectively).

The mean number of prescribed medications was statistically higher in the treatment group compared to the control group (*p* ≤ 0.001). This difference remained statistically significant even after excluding the SGLT2 inhibitor from the total count (*p* ≤ 0.05). Polypharmacy (defined as ≥5 medications) was significantly higher in the treatment group compared to the control group (80% vs. 60%; *p* ≤ 0.05).

### 3.2. Changes in Clinical Parameters During Follow-Up and Safety Outcomes

The patients treated with SGLT2I demonstrated statistically significantly greater mean reductions compared to the control group in weight, BMI, FPG, HbA1c, SBP, and DBP (*p* ≤ 0.01 for FPG and DBP and *p* ≤ 0.001 for the rest of the parameters). Regarding the relative reduction in HbA1c, the mean percentage decrease was significantly greater in the treatment group (−13.06 ± 11.35%) compared to the control group (−8.50 ± 7.65%) (*p* ≤ 0.01) (Table 3).

When HbA1c values were evaluated categorically, the treatment group exhibited a less favorable status at baseline, with fewer patients having a tight glycemic goal (HbA1c < 6.5%) and a higher proportion of patients having poor control (HbA1c > 7.5%) compared to the control group (*p* < 0.001). However, no significant inter-group difference was observed in the HbA1c categorical distribution at the subsequent follow-up (*p* > 0.05). Notably, intra-group analysis within the treatment group demonstrated significant improvement over time: the number of patients achieving HbA1c < 6.5% increased significantly, while the number of patients with HbA1c > 7.5% decreased significantly between the first and subsequent follow-ups (*p* < 0.001 for both changes).

At baseline, the treatment group had statistically significantly lower HDL cholesterol (44.03 ± 11.55 vs. 48.92 ± 12.71; *p* ≤ 0.01) and higher triglyceride levels (212.30 ± 179.57 vs. 168.10 ± 85.95; *p* ≤ 0.05) compared to the control group, while total and LDL cholesterol levels were similar (*p* > 0.05). Following the treatment period, the treatment group showed significantly lower TG levels compared to the baseline (*p* ≤ 0.05). Lipid profile is detailed in Table 4.

In the intra-group analysis, serum creatinine levels significantly decreased in the treatment group from baseline to final follow-up (0.88 ± 0.26 vs. 0.84 ± 0.27 mg/dL, *p* = 0.008). In contrast, no statistically significant change was observed in the control group (0.878 ± 0.36 vs. 0.89 ± 0.41 mg/dL, *p* = 0.542). When comparing the categorical eGFR distribution between the treatment group and the control group, no statistically significant difference was observed at the first follow-up or at the subsequent follow-up time point (*p* > 0.05 for both comparisons) (Table 5).

No statistically significant differences were observed between the treatment group and the control group regarding the prevalence of all reported adverse events. In addition, none of the patients developed euglycemic ketoacidosis and Fournier’s gangrene. The proportion of patients with glucosuria was statistically significantly higher in the treatment group at both time points compared to the control group (*p* ≤ 0.001 for both). Specifically, at the first follow-up, 64% of SGLT2I patients had glucosuria versus 14% of patients in the control group, and this difference widened at the subsequent follow-up (86% vs. 10%). Similarly, urine density was found to be significantly higher in the treatment group than in the control group at both the first and subsequent follow-ups (*p* ≤ 0.001). In a sub-group analysis within the treatment group, patients with glucosuria showed a statistically significantly higher mean urine density (1.026 ± 0.009) compared to those without glucosuria (1.018 ± 0.007) (*p* ≤ 0.01).

### 3.3. Multivariable Regression Analyses of HbA1c, Body Weight, and Blood Pressure

Multivariable linear regression analyses were performed for all primary continuous outcomes. In the primary multivariable model, using absolute HbA1c change as the outcome and adjusting for baseline HbA1c, age, sex, insulin, biguanide, and DPP4i use, the SGLT2I treatment effect showed borderline significance (β = 0.253, 95% CI: −0.03 to 0.53, *p* = 0.077). In the sensitivity analysis using percentage HbA1c change as the outcome, SGLT2I treatment was a significant independent predictor of glycemic improvement (β = 5.81, 95% CI: 1.81 to 9.80, *p* = 0.004). After adjustment for age, sex, baseline weight, and insulin use, SGLT2I treatment was a significant independent predictor of body weight reduction (β = 2.52 kg, 95% CI: 0.88 to 4.16, *p* = 0.003). In patients with available blood pressure measurements (*n* = 120), after adjustment for sex, baseline SBP, beta-blocker, and diuretic use, SGLT2I treatment was an independent predictor of systolic blood pressure reduction (β = 10.87 mmHg, 95% CI: 4.21 to 17.53, *p* = 0.002). The independent effect on diastolic blood pressure did not reach statistical significance (β = 4.97, 95% CI: −0.41 to 10.35, *p* = 0.070) (Table 6).

## 4. Discussion

In this study, treatment with SGLT2 inhibitors was associated with statistically significantly greater mean reductions in multiple cardiometabolic parameters compared to the patients treated without SGLT2I. Specifically, the patients treated with SGLT2I showed superior reductions in HbA1c, FPG, weight, BMI, SBP, and DBP.

In our study, there was a significant reduction in mean FPG (−24.2 mg/dL) and HbA1c (−0.76%) values in the treatment group. A recent meta-analysis, primarily aimed at evaluating the impact of SGLT2I on the components of metabolic syndrome (MetS), reported a significant glycemic reduction with a mean decrease in FPG of −18.07 mg/dL (95% CI: −25.32 to −10.82) and HbA1c of −0.68% (95% CI: −0.88 to −0.48) in the SGLT2I-treated group compared to the placebo [18].

In addition, although mean FPG and HbA1c values were significantly higher in the treatment group at baseline compared to the control group, the number of patients whose HbA1c values decreased and who reached the target HbA1c value at the end of follow-up was significantly higher in the treatment group compared to the control group.

The absence of a significant difference between the groups in insulin and non-insulin antidiabetic drugs and drugs that may affect the glycemic values and metabolic syndrome of the patients supports the fact that the glycemic benefit seen in the treatment group is directly related to the use of SGLT2I.

On the other hand, the treatment group was statistically younger at baseline than patients in the control group. In a network meta-analysis investigating the effect of age and sex on the efficacy of SGLT2I treatment of patients with T2DM, the use of SGLT2I (vs. placebo) was associated with less HbA1c lowering with increasing age for monotherapy (absolute reduction [AR], 0.24% [95% credible interval [19], 0.10% to 0.38%] per 30-year increment in age), for dual therapy (AR, 0.17% [95% CrI, 0.10% to 0.24%]), and for triple therapy (AR, 0.25% [95% CrI, 0.20% to 0.30%]) [20]. This age difference presents a clear confounding factor in interpreting HbA1c findings.

SGLT2I’s primary advantage is related to modulating key cardiometabolic risk factors, specifically body weight reduction and blood pressure reduction. In this study, there was a significant reduction in mean body weight (−3.6 kg) and calculated BMI (−1.2 kg/m^2^) with SGLT2Is. The weight loss advantage of the SGLT2I in our study is consistent with previous RCTs and meta-analytic evidence. For instance, patients on empagliflozin experienced weight reductions of up to −2.2 kg over 78 weeks, in contrast to the weight gain observed in the placebo group [21]. Similarly, the dapagliflozin trial found that SGLT2I led to sustained weight loss of approximately −2.43 kg over 48 weeks, effectively neutralizing the mean weight gain that occurred in the high-dose insulin-only group [22]. Consequently, SGLT2Is may successfully mitigate the weight gain typically associated with insulin treatment while improving glycemic control.

A network meta-analysis by Pinto et al. [19] showed that SGLT2Is significantly reduce weight compared to sulfonylureas, reporting a mean difference of −4.76 kg in favor of SGLT2I. Similarly, Vasilakou et al. [23] demonstrated broad comparative efficacy, with SGLT2I reducing body weight by a mean difference of −1.11 kg (95% CI: −1.46 kg to −0.76 kg) when compared against other established agents, including metformin, sitagliptin, and sulfonylureas.

In our study, significant decreases in systolic (−9.2 mmHg) and diastolic blood pressure (−4.7 mmHg) were observed in the treatment group. SGLT2Is show greater effectiveness in lowering SBP and DBP compared to metformin, sulfonylureas, and DPP4 inhibitors. The meta-analysis, comparing SGLT2I to metformin, sitagliptin, and sulfonylureas, showed a mean reduction in SBP of −4.45 mm Hg and a mean reduction in DBP of −2.01 mm Hg [23]. Similarly, Pinto et al. [19] reported that SGLT2I treatment resulted in markedly superior BP lowering against metformin (SBP −5.86 mmHg), SU (SBP −5.44 mmHg; DBP −2.59 mmHg), and DPP4i (SBP −4.43 mmHg; DBP −1.89 mmHg), emphasizing their unique advantage in cardiovascular risk management beyond glycemic control. The BP reduction in our cohort was approximately twofold greater than typically reported in the literature, suggesting a highly exaggerated effect in this population. This finding is likely compounded by the statistically significant higher use of beta-blockers within the SGLT2I group, as these co-prescribed anti-hypertensive agents would naturally amplify the overall BP-lowering outcome.

Hu et al. [24] suggested that weight reduction was significantly and independently associated with SBP reduction (Beta = 0.820; 95% CI, 0.332 to 1.307; *p* = 0.001) but was not significantly associated with DBP reduction (Beta = 0.268; 95% CI, −0.019 to 0.556; *p* = 0.067). Weight loss may be a key mechanism of action for the SBP reduction observed with SGLT2I, while other weight-independent effects, such as osmotic diuresis, may be more dominant for DBP. Moreover, Diallo et al. [25] reported that every 5 mmHg reduction in SBP was associated with a significantly lower risk of MACE (HR 0.81; 95% CI: 0.74 to 0.89) and HF (HR 0.49; 95% CI: 0.42 to 0.57). Furthermore, a sub-group analysis focusing on the interaction between BP reduction and weight loss suggested that the benefit of SGLT2I’s BP-lowering effect on stroke (which was otherwise non-significant) was specifically tied to the magnitude of greater body weight reduction (<−1.5 kg) (P_interaction_ = 0.0177).

To address the baseline imbalance between groups, multivariable regression analyses were performed for all primary outcomes. Regarding glycemic outcomes, two complementary analytical approaches were employed. In the primary adjusted model using absolute HbA1c change, the SGLT2I treatment effect showed borderline significance (β = 0.253, *p* = 0.077), likely reflecting the strong regression-to-mean effect driven by the significantly higher baseline HbA1c in the SGLT2I group. A sensitivity analysis using percentage HbA1c change, which inherently normalizes for baseline values, confirmed a significant independent treatment effect (β = 5.81, *p* = 0.004), supporting the conclusion that the observed glycemic benefit is attributable to SGLT2 inhibitor therapy.

Despite the significant baseline differences in age, sex, cardiovascular comorbidities, and metabolic parameters, SGLT2I treatment remained an independent predictor of body weight reduction (β = 2.52 kg, *p* = 0.003) and systolic blood pressure reduction (β = 10.87 mmHg, *p* = 0.002) after covariate adjustment. These findings strengthen the conclusion that the observed cardiometabolic benefits are attributable to SGLT2I therapy rather than confounding alone. The independent effect of SGLT2I on diastolic blood pressure did not reach statistical significance in the adjusted model (β = 4.97, *p* = 0.070). This finding is also consistent with the known differential effects of SGLT2I on systolic versus diastolic blood pressure reported in the literature, where systolic reductions are consistently larger and more robust than diastolic effects [24]. The absence of a significant independent DBP effect in our adjusted model may reflect a true pharmacological pattern rather than a study-specific limitation.

In our study, the majority of patients in the treatment group were using empagliflozin. Patients with T2DM who had known cardiovascular disease or were at high risk showed that while empagliflozin and canagliflozin demonstrated a reduction in MACE, dapagliflozin reduced the incidence of cardiovascular death irrespective of a history of atherosclerotic cardiovascular disease but did not significantly reduce MACE [26,27,28]. The high prevalence of empagliflozin prescription within our patient cohort may be attributable to its superiority over dapagliflozin in the reduction in MACE risk.

The inclusion of SGLT2Is in guidelines for the treatment of diabetic patients with atherosclerotic cardiovascular disease and heart failure [29], due to their cardiac benefits and their preference in patients with these comorbidities, could explain in our study why the number of patients with these comorbidities in the treatment group was significantly higher compared to the control group.

A recent meta-analysis [30], including over 147,000 participants, reported that SGLT2 inhibitors significantly increase total, LDL, and HDL cholesterol, while significantly lowering triglycerides. In our study, no statistically significant changes were observed in total or LDL cholesterol in the treatment group. This may be due to factors such as the tighter monitoring of patients in the treatment group compared to the control group, the greater weight loss in these patients, or the greater use of lipid-lowering medications in the treatment group. In our study, serum HDL levels measured in the treatment group were significantly lower than in the control group at both the first and subsequent follow-ups, while serum TG levels were initially higher than in the control group. Women have been shown to have higher serum HDL levels and lower TG levels than men [31]. The significantly higher number of male patients than female patients in the treatment group in our study may have influenced serum HDL and TG levels. The significant decrease in serum TG level in the treatment group at subsequent follow-up was consistent with other studies.

The cardiometabolic benefits observed in our study are consistent with findings from large real-world observational programs. The CVD-REAL and OBSERVE-4D studies demonstrated that SGLT2 inhibitors significantly reduce the risk of hospitalization for heart failure and mortality [32,33]. Furthermore, the EMPRISE study showed that empagliflozin reduces not only heart failure hospitalization but also the risk of major adverse cardiovascular events, including myocardial infarction and stroke [34]. Beyond glycemic and cardiovascular outcomes, growing evidence highlights the pleiotropic potential of SGLT2 inhibitors. For example, a recent network meta-analysis by Mariani et al. found that these agents, particularly dapagliflozin, significantly reduce the risk of atrial fibrillation [35].

Given that cardiometabolic parameters such as hypertension, obesity, and poor glycemic control are key modifiable risk factors for atrial fibrillation, these findings suggest that the benefits of SGLT2 inhibitors may extend well beyond heart failure and diabetes management. The convergence of these large real-world datasets with our findings further confirms that the cardiometabolic benefits of SGLT2 inhibitors are robust and clinically meaningful in routine clinical practice.

In our study, a statistically significant decrease in serum creatinine levels was observed in the treatment group from baseline to follow-up. While this finding may suggest a favorable trend in renal function, it should be interpreted with caution given the retrospective design and relatively short follow-up period. A recent pharmacovigilance study suggested that SGLT2 inhibition may enhance creatinine clearance through mechanisms related to renal glucose excretion, which could partly explain the reduction observed in our cohort [36].

In our study, the significant decrease in serum creatinine levels observed in the treatment group suggests an improvement in renal filtration capacity. This finding is supported by a recent pharmacovigilance and pharmacokinetic study by Uwai et al. [36], which identified a strong association between SGLT2 inhibitor use and increased creatinine clearance in the FDA Adverse Event Reporting System (FAERS). The authors demonstrated in animal models that SGLT2 inhibition enhances creatinine clearance, strongly correlating with renal glucose excretion [36]. This suggests that the glucosuria induced by SGLT2 inhibitors may physiologically contribute to the augmented creatinine clearance and the subsequent reduction in serum creatinine levels we observed.

In our study, there were no patients with end-stage renal disease in either group. No significant difference was observed in the GFR values calculated at subsequent follow-up in the treatment group. There was a significant decrease in serum creatinine level in the treatment group at the subsequent follow-up compared to the first follow-up. A meta-analysis of over 70,000 participants established a significant and multifaceted renoprotective effect of the SGLT2I class. SGLT2I significantly reduced the risk of the composite renal outcome and several hard renal endpoints, including sustained eGFR decline, doubling of serum creatinine, and progression to ESRD or dialysis. This protective action also included a reduced risk of acute kidney injury [37].

Similarly, another meta-analysis (58,534 participants) showed an acute, temporary reduction in eGFR during the initial 2–4 weeks of treatment as related to renal hemodynamic effects. Crucially, this was followed by a long-term renoprotective effect, characterized by a significantly slower rate of eGFR decline compared to placebo. This sustained benefit became statistically evident around the two years of treatment, confirming that SGLT2I effectively slows the progression of kidney function decline across various CKD stages, regardless of diabetes status [38]. Although our results show consistency with the renoprotective effects reported in large-scale meta-analyses, the retrospective design, limited follow-up, and the younger mean age and better baseline renal function of patients in the SGLT2I group preclude definitive conclusions regarding long-term renal outcomes. Prospective studies with longer follow-up are needed to confirm these findings.

Overall, SGLT2 inhibitors exhibit a favorable and well-tolerated safety profile [39,40]. Due to their insulin-independent mechanism of action, they are associated with a minimal risk of hypoglycemia compared to other antidiabetic agents [39,40]. While drug-induced glycosuria significantly increases the incidence of genital mycotic infections, these common adverse events are generally mild to moderate and can be easily managed with standard antimicrobial therapies without requiring treatment discontinuation [41]. Although rare, clinical vigilance is recommended for potentially severe complications such as euglycemic diabetic ketoacidosis and hypovolemia [40].

In our study, the significantly higher rate of glucosuria in the treatment group was a natural result of SGLT2I’s mechanism of action. Interestingly, there was an increase in urine density in the treatment group compared to the control group, which is consistent with findings from an observational study [42]. When we compared the urine densities of patients with and without glucosuria in the treatment group, the urine density was higher in patients with glucosuria. Consequently, there may be a correlation between glucosuria and urine density.

The findings of this study identify important directions for future research. While our results underscore the cardiometabolic benefits of SGLT2 inhibitors in a general T2DM outpatient cohort, further investigation is warranted in high-risk sub-groups. Specifically, prospective studies focusing on the integrated effects of SGLT2 inhibitors on cardiometabolic parameters and renal outcomes in patients with comorbid heart failure or coronary artery disease would provide essential evidence for these clinically complex populations. As highlighted by Ciuca-Pană et al. [43], the pharmacological management and cardiac rehabilitation of such patients remain a dynamic field; thus, the specific role of SGLT2 inhibitors requires dedicated exploration within standardized rehabilitation programs to mitigate lifestyle biases and accurately evaluate therapeutic efficacy.

### Limitations

Our study has several limitations. First, the follow-up period in our study was sufficient, but it may be relatively short for assessing long-term outcomes. Second, missing data, particularly for BP measurements, hindered some analyses. Third, the study was conducted during the COVID-19 pandemic, which may have affected hospital admissions and patient management. Finally, the retrospective nature prevents the establishment of definitive cause-and-effect relationships.

## 5. Conclusions

SGLT2Is, with their ability to improve glycemic control, reduce cardiovascular risk factors, and provide renoprotective benefits, may be considered first-line medication, particularly for diabetic patients with poor glycemic profiles and comorbidities. Robust, long-term real-world studies are necessary to further confirm these safety and efficacy profiles.

## Figures and Tables

**Table 1:** Baseline characteristics of the patients.

Characteristic	Treatment Group (*n* = 100)	Control Group (*n* = 100)	*p*-Value
**Demographic Data, Mean ± SD**			
Age (years)	55.86 ± 10.88	58.94 ± 10.03	≤0.05
Age Categories, %			
<65 years	80	65	≤0.05
≥65 years	20	35	
Gender, *n* (%)			
Male	55	34	≤0.01
Female	45	66	
Weight (kg)	89.14 ± 17.68	84.71 ± 17.92	>0.05
BMI (kg/m^2^)	32.16 ± 6.26	31.79 ± 6.47	>0.05
**Clinical Data, Mean ± SD**			
Fasting blood glucose (mg/dL)	159.54 ± 60.97	139.10 ± 55.26	≤0.05
HbA1c (%)	7.92 ± 1.61	7.00 ± 1.41	≤0.001
SBP (mmHg) *	134.99 ± 19.93	129.70 ±18.48	>0.05
DBP (mmHg) *	81.18 ± 10.94	77.64 ± 11.01	>0.05
Creatinine (mg/dL)	0.88 ± 0.26	0.878 ± 0.36	>0.05
Urea (mg/dL)	33.51 ± 13.87	32.30 ± 14.35	>0.05
**CKD Stage Based on eGFR (mL/dk/1.73m^2^), %**			
eGFR ≥ 60	87	85	>0.05
30 < eGFR ≤ 59	13	14	
20 < eGFR ≤ 30	-	1	
**Comorbidities, %**			
Atherosclerotic cardiovascular disease	39	15	≤0.001
Coronary artery disease	35	11	≤0.001
Cerebrovascular disease	8	5	0.390
Myocardial infarction	11	0	≤0.001
Peripheral arterial disease	4	2	0.407
Heart failure	19	4	≤0.001
Hypertension	60	66	0.380
Diabetic foot disease	2	4	0.407
Chronic kidney disease	15	15	0.684

Abbreviations: BMI, body mass index; SBP, systolic blood pressure; DBP, diastolic blood pressure. * SBP and DBP were not available for all patients and were measured in 67 patients in the SGLT2I group and 53 patients in the non-SGLT2I group.

**Table 2:** Prevalence of medication at baseline.

Medications	Treatment Group (*n* = 100)	Control Group (*n* = 100)	*p*-Value
**Glucose-lowering therapies, %**			
Metformin	84	87	0.547
Insulin	47	37	0.152
Sulfonylurea	6	8	0.579
DPP-4 inhibitor	42	49	0.320
Thiazolidinedione	6	7	0.774
GLP-1 agonist	10	10	1.000
Meglitinides	4	1	0.174
α-Glucosidase inhibitors	0	3	0.081
**Anti-hypertensive therapy, %**	74	75	0.871
Beta-blockers	46	26	≤0.01
ACE inhibitors/ARBs	60	60	1.000
Diuretics	33	38	0.460
Calcium channel blockers	19	31	≤0.05
**Lipid-lowering therapy, %**	64	41	≤0.001
**Antithrombotic therapy,%**	48	24	≤0.001
**PPIs, %**	25	18	0.228
**Antiepileptic, %**	8	5	0.390
**Antidepressant, %**	13	19	0.247
**Antipsychotic, %**	5	7	0.552
**Osteoporosis medications, %**	5	2	0.651
**Steroid, %**	1	1	1.000

Abbreviations: DPP-4, dipeptidyl peptidase-4; GLP-1, glucagon-like peptide-1; ACE, angiotensin-converting enzyme; ARB, angiotensin receptor blockers; PPIs, proton pump inhibitors.

**Table 3:** Follow-up outcomes and adverse events in the SGLT2I-treated and control groups.

Characteristic	Treatment Group (*n* = 100)	Control Group (*n* = 100)	*p*-Value (Inter-Group)
**Demographic Data, Mean Change ± SD**			
Weight (kg)	−3.59 ± 5.70	−0.58 ± 5.15	≤0.001
BMI (kg/m^2^)	−1.24 ± 2.09	−0.22 ± 1.93	≤0.001
**Clinical Data, Mean Change ± SD**			
Fasting blood glucose (mg/dL)	−24.24 ± 58.98	−6.27 ± 44.66	≤0.05
HbA1c (%)	−0.758 ± 1.33	−0.093 ± 0.89	≤0.001
SBP (mmHg) *	−9.16 ± 22.59	5.77 ± 22.01	≤0.001
DBP (mmHg) *	−4.72 ± 13.45	2.17 ± 18.06	≤0.05
**Incidence of Adverse Events, %**			
Genitourinary infections	12	7	0.228
Polyuria	11	8	0.469
Polydipsia	7	8	0.788
Fatigue	15	14	0.841
Dizziness	7	2	0.088
Arthralgia	12	9	0.489
Osteoporosis	1	3	0.312
Diabetic foot events	1	0	0.316
Euglycemic ketoacidosis	0	0	-
Fournier’s gangrene	0	0	-

Abbreviations: BMI, body mass index; SBP, systolic blood pressure; DBP, diastolic blood pressure. * SBP and DBP were not available for all patients and were measured in 67 patients in the SGLT2I group and 53 patients in the non-SGLT2I group.

**Table 4:** Lipid profiles of treatment and control groups.

	Treatment Group (*n* = 100)	Control Group (*n* = 100)
**Total Cholesterol**		
Baseline	187.90 ± 49.51	190.02 ± 40.28
Follow-up	180.08 ± 39.61	187.64 ± 38.31
**LDL**		
Baseline	104.75 ± 40.11	109.23 ± 34.08
Follow-up	100.99 ± 31.21	107.84 ± 31.47
**HDL**		
Baseline	44.03 ± 11.55 ^a^	48.92 ± 12.71
Follow-up	43.67 ± 10.78 ^b^	48.02 ± 13.36
**TG**		
Baseline	212.30 ± 179.57 ^c^	168.10 ± 85.95
Follow-up	190.25 ± 167.88 ^d^	161.85 ± 90.16

^a^, ^b^, ^c^ treatment vs. control; ^d^ baseline vs. follow-up: *p* ≤ 0.05.

**Table 5:** Renal parameters of treatment and control groups.

	Treatment Group (*n* = 100)	Control Group (*n* = 100)
**Creatinine**		
Baseline	0.88 ± 0.26	0.878 ± 0.36
Follow-up	0.84 ± 0.27 ^a^	0.89 ± 0.41
**Urea**		
Baseline	33.51 ± 13.87	32.30 ± 14.35
Follow-up	33.45 ± 15.18	33.17 ± 17.41
**CKD Stage Based on eGFR (mL/dk/1.73m^2^), %**		
Baseline		
eGFR ≥ 60	87	85
30 < eGFR ≤ 59	13	14
20 < eGFR ≤ 30	0	1
Follow-up		
eGFR ≥ 60	87	83
30 < eGFR ≤ 59	13	14
20 < eGFR ≤ 30	0	3

^a^ baseline vs. follow-up: *p* ≤ 0.05.

**Table 6:** Multivariable regression analysis: effect of SGLT2 inhibitor treatment on cardiometabolic outcomes.

	Analysis	β (SGLT2I)	SE	95% CI	*p*-Value	Covariates Adjusted
HbA1c % change	Sensitivity	5.807	2.037	1.81, 9.80	**0.004**	Age, sex, insulin, biguanide, DPP4i
HbA1c absolute change (%)	Primary adjusted	0.253	0.142	−0.03, 0.53	0.077	Age, sex, baseline HbA1c, insulin, biguanide, DPP4i
Body weight change (kg)	Primary adjusted	2.520	0.829	0.88, 4.16	**0.003**	Age, sex, baseline weight, insulin
SBP change (mmHg)	Primary adjusted	10.869	3.364	4.21, 17.53	**0.002**	Sex, baseline SBP, beta-blocker, diuretic
DBP change (mmHg)	Primary adjusted	4.974	2.715	−0.41, 10.35	0.070	Sex, baseline DBP, beta-blocker, diuretic

Abbreviations: SE, standard error; CI, confidence interval; SBP, systolic blood pressure; DBP, diastolic blood pressure; DPP4i, dipeptidyl peptidase-4 inhibitor. Notes: β coefficients represent the independent effect of SGLT2I vs. non-SGLT2I treatment. Positive β for blood pressure outcomes indicates greater reduction in the SGLT2I group relative to control (reference: lower baseline). HC3 heteroscedasticity-robust standard errors used throughout. BP analyses restricted to patients with available measurements (*n* = 120). Sensitivity analysis for HbA1c uses percentage change to adjust for baseline imbalance.

## Data Availability

The datasets used and/or analyzed during the current study are available from the corresponding author on reasonable request.
